# Detection of human MCP-4/CCL13 isoforms by SELDI immunoaffinity capture

**DOI:** 10.1186/1479-5876-4-5

**Published:** 2006-01-24

**Authors:** Leonardo Rossi, Ramy Moharram, Brian M Martin, Richard L White, Monica C Panelli

**Affiliations:** 1 Department of Human Morphology and Applied Biology, University of Pisa, 56126 Pisa, Italy; 2 National Institute of Mental Health NIMH, National Institutes of Health, Bethesda, Maryland 20892 USA; 3 Carolinas Medical Center, Blumenthal Cancer Center, Charlotte, North Carolina, 28232 USA; 4 Immunogenetics Section, Department of Transfusion Medicine, Clinical Center, National Institutes of Health, Bethesda, Maryland 20892, USA

## Abstract

Monocyte Chemoattractant Proteins 4 (MCP-4/CCL13) is a member of a distinct, structurally-related subclass of CC chemokines mainly involved in recruitment of eosinphils to inflammatory sites. Recent evidence demonstrates that serum level of this protein strongly increases following high dose IL-2 immunotherapy. The physiological form of human MCP-4/CCL13 has yet to be purified. Therefore, the primary structure of the biologically relevant (mature) form has not been established. By using SELDI immunoaffinity capture technology we describe two mature isoforms both present in serum before and after high-dose IL-2 immunotherapy.

## Introduction

Monocyte Chemoattractant Proteins 4 (MCP-4/CCL13) is member of a distinct, structurally-related subclass of CC chemokines. MCP-4/CCL13 is a major chemoattractants for eosinophils, basophils monocytes and T lymphocytes. The MCP protein family bind to specific G-protein-coupled receptors, initiating a signal cascade within the cell [1,2]. Expression of MCP-4/CCL13 mRNA and protein is greater in the sputum, epithelium, submucosal inflammatory cells and bronchoalveolar lavage fluid of asthmatics than in healthy individuals [3,4]. Involvement of MCPs has also been demonstrated in renal inflammation and atopic dermatitis [5,6] and the expression of this protein is also correlated with enhanced inflammatory immune responses during immunotherapy. Specifically, massive production of chemoattractants, has been demonstrated by quantitative methods (multiplex protein array/Searchlight) in renal cell carcinoma (RCC) patient serum after high dose IL-2 therapy [7].

Our qualitative analysis of MCP-4/CCL13 was based on SELDI immunocapture. We and others have previously used this proteomic tool to rapidly screen and differentiate, based on mass/charge (m/z) ratios isoforms of serum proteins [8,9]. This type of approach represents an integral step in the detection of biologically relevant differences among systemic proteins during physiological and pathological states. It also aids in the biomarker discovery process associated with a disease state or the occurrence of a vigorous immune response such as that elicited by high dose IL-2. SELDI spectra may in fact suggest the presence of specific post-translational modifications such as glycosylation or phosphorylation at specific sites, early protein truncation and other events [10] that may define a specific isoform and thus constitute a biomarker.

The aim of this study was to qualitatively characterize MCP-4/CCL13 by Surface Enhanced Laser Desorption Ionization Time Of Flight Mass Spectrometry (SELDI-TOF-MS™) in the serum of RCC patients undergoing high dose IL-2 immunotherapy. We were also able to detect, for the first time in human serum two systemic MCP-4/CCL13 isoforms of similar molecular weight before and after IL-2 treatment.

## Materials and methods

### Patients and serum collection

Ten patients with metastatic RCC were recruited at the Carolinas Medical Center (Charlotte, NC) to receive systemic high-dose (720,000 IU/Kg every 8 hrs) IL-2 administration (Proleukin, Chiron, Emeryville, CA). Serum was collected prior to IL-2 treatment (PRE) and 3 hours after the fourth (POST 4) dose a time point that corresponds to the clinical observation of its peak effects (three to four hours after administration of intravenous bolus IL-2) [7,8], aliquoted and stored at -80°C until testing. All tested aliquots were subjected to only one freeze-thaw cycle.

### Immunoprecipitation

Five hundred micrograms of MCP-4/CCL13 antibody clone 18 (Santa Cruz Biotechnology Santa Cruz, CA) were bound to ImmunoPure Immobilized Protein G column according to the manufacturer protocol (Seize X Protein G Immunoprecipitation Kit, Pierce, Rockford, IL) for 1 hour at room temperature. The bound antibody was crosslinked to protein G using disuccinimidyl suberate (DSS) reagent. After quenching the reaction (washing multiple times with ImmunoPure^® ^IgG elution buffer pH 2.8 containing primary amine), beads were equilibrated in the column with binding/washing buffer (0.14 M NaCl, 0.008 M sodium phosphate, 0.002 M potassium phosphate and 0.01 M KCl, pH7.4. One milliliter of serum was diluted 1:1 with binding/washing buffer and incubated with the resin containing the immobilized antibody on a roller platform for five hours at room temperature. Twenty microliters of halt protease inhibitor cocktail, EDTA free (Pierce) were added to inhibit proteases and prevent protein degradation.

Unbound proteins were removed by centrifugation and a fraction of the flow-through was saved for further analysis.

Proteins bound to the column were eluted with 200 μl of elution buffer and neutralized by adding 10 μl of 1 M tris-HCl. The eluted proteins were stored at -80°C for future analysis.

### ProteinChip immunoaffinity capture

Immunoaffinity capture was performed using a PG20 Array (ProteinChip^® ^antibody capture kit, Ciphergen Biosystems, Fremont, CA, USA). Polyclonal anti-MCP-4/CCL13 antibody clone 18 and clone 20 (Santa Cruz Biotechnology Santa Cruz, CA), and Polyclonal anti IL-6 antibody (Abcam, Cambridge, MA) were used at a final concentration of 0.2 μg/μl in PBS. These affinity purified goat polyclonal antibodies were raised against a peptide mapping near the carboxy terminus of MCP-4 of human origin. Protein G arrays were placed in a bioprocessor (Ciphergen Biosystems) and rehydrated in PBS for 1 min before antibody binding to protein G. The capture of MCP-4/CCL13 protein was performed by placing 20 μl of the appropriate antibody on a single spot of the array followed by incubation for 90 min at room temperature on a shaker. Unbound antibodies were removed by washing twice with 400 μl of wash buffer (0.5% (v/v) Triton X100 in PBS) and twice with PBS. Bound antibodies were cross-linked to protein G by adding 30 μl of bis(sulfosuccinimidyl) suberate (supplied in the ProteinChip antibody capture kit) at a final concentration of 0.5 mg/ml for 30 min at room temperature. The residual active sites were finally deactivated by adding 30 μl of 0.5 M ethanolamine in PBS pH 8. After a 15 min incubation each spot was washed twice with 400 ul of wash buffer and twice with PBS. Twenty μl of samples diluted 1:1 with PBS were subsequently placed on the spots. Chips were incubated for 3 hours at room temperature, washed twice with 400 ul of wash buffer and twice with PBS, rinsed in 1 M HEPES buffer and air dried. Prior to SELDI-TOF MS analysis, 0.6 μl of saturated sinapinic acid (SPA) solution was added to each spot.

### Surface Enhanced Laser Desorption/Ionization – Time of Flight Mass Spectroscopy (SELDI-TOF MS)

All mass spectra were recorded in the positive-ion mode on the SELDI mass spectrometer PBS IIc ProteinChip Array reader, a linear laser desorption/ionization time-of-flight mass spectrometer with time-lag focusing [14]. Raw data were analyzed using computer software provided by the manufacturer and are reported as average masses.

## Results

### Identification of MCP-4/CCL13 isoforms in sera from RCC patient undergoing high dose IL-2 treatment

**Figure 1:**
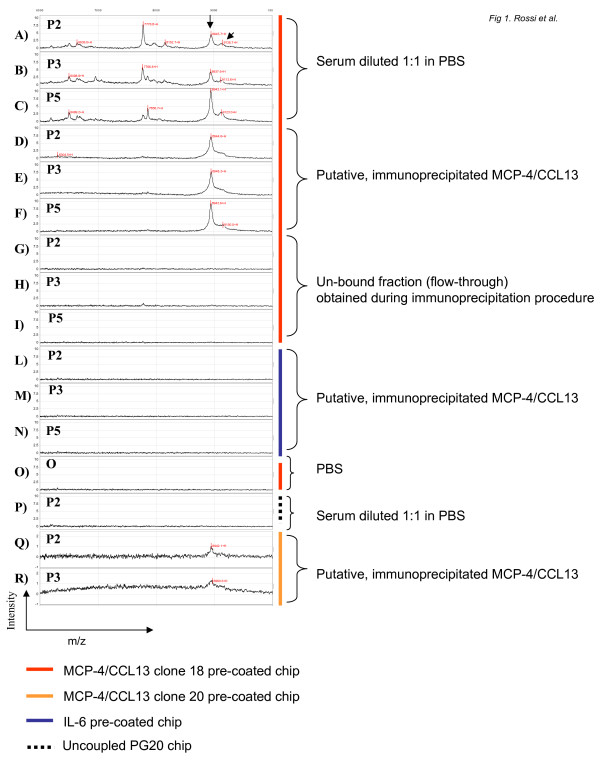
SELDI immunoaffinity capture of MCP-4/CCL13 from patient sera after high dose IL-2 immunotherapy. Anti MCP-4/CCL13 clone 18 (A-I, O), anti MCP-4/CCL13 clone 20 (Q,R) and anti IL-6 (L-N) polyclonal antibody were immobilized on a protein G array. Spectra from 6000 to 10000 Da are depicted for each experiment. No significant peaks were detected out of this range. Immunoaffinity capture spectra of patient 2(A), 3(B), 5(C) serum diluted 1:1 in PBS. Immunoaffinity capture spectra of MCP-4/CCL13 immunoprecipitated from patient 2(D), 3(E), 5(F). (G) Immunoaffinity capture spectra of the un-bound fraction (flow-through) obtained during the immunoprecipitation procedure of patient 2(G), 3(H), 5(I). Immunoaffinity capture spectra of MCP-4/CCL13 immunoprecipitated from patient 2(L), 3(M), 5(N) serum on IL-6 pre-coated chip. (O) Immunoaffinity capture spectra of PBS. (P) spectra of patient 2 serum applied on an uncoupled PG20 chip. Immunoaffinity capture spectra of MCP-4/CCL13 immunoprecipitated from patient 2(Q) and 3(R) serum on MCP-4/CCL13 clone 20 pre-coated chip. Intensity scale for A and P spectra = 0 – 10. Intensity scale for Q and R = 0 – 2. Red bar = MCP-4/CCL13 clone 18 pre-coated chip; orange bar = MCP-4/CCL13 clone 20 pre-coated chip; blue bar = IL-6 pre-coated chip; dotted bar = Uncoupled PG20 chip.

The use of SELDI-TOF-MS combined with specific antibodies raised against MCP-4/CCL13 protein allowed us to identify a dominant peak at about 8945 m/z and a second peak at about 9113 m/z that approximately matched with MCP-4/CCL13 putative molecular weight calculated from the amino acid sequence. These peaks were especially prominent when straight serum obtained after the 4th dose of IL-2 was applied on PG20 chip pre-coated with anti MCP-4/CCL13 clone 18 antibody (Fig 1A-C: spectra from 3 representative patients P2, P3 and P5). We immunoprecipitated MCP-4/CCL13 prior to immunocapture on the chip surface in order to establish the specificity of the detection as well as reduce the background from contaminating proteins. One ml of serum was immunoprecipitated with anti MCP-4/CCL13 clone 18 antibody and the eluted proteins were applied on PG20 chips pre-coated with the same antibody. A peak at about 8945 m/z accompanied by a smaller peak at about 9113 m/z was detected in all the samples (Fig 1D-F: spectra from patient P2, P3 and P5). The unbound fraction (flow-through, see materials and methods for details) obtained during the immunoprecipitation procedure did not show any specific binding when applied on MCP-4/CCL13 clone 18 pre-coated PG20 chips (Fig 1G-I: spectra from patient P2, P3 and P5) confirming the specificity of our detection. No peaks were obtained when the immunoprecipitated fraction was applied on PG20 chips pre-coated with the unrelated anti IL-6 antibody (Fig 1L-N: spectra from patient P2, P3 and P5). No peaks were detected in chips loaded with negative controls (PBS applied on MCP-4/CCL13 pre-coated chips Fig 1O, or crude serum applied on PG20 chip without pre-coating, Fig 1P). To further confirm the specificity of detection of MCP-4/CCL13, the immunoprecipitated fraction was applied to PG20 chips pre-coated with a different clone of anti MCP-4/CCL13 antibody (clone 20) The aforementioned 8945 peak was also detected by this antibody clone (Fig 1Q,R: spectra from patient P2, P3) in the pre and post samples. SELDI immunocapture technology allowed us to detect MCP-4/CCL13 protein in serum samples obtained prior to IL-2 treatment; no differences in the expression level between pre and post samples were detectable using this technology in our experimental condition (data not shown).

## Discussion

Novel mass spectrometry based technologies, such as SELDI-TOF-MS, have shown promising results especially when combined with biospecific interactive surfaces. The use of specific antibodies applied on the SELDI chip surface is a powerful tool to selectively identify a desired target in a complex protein mixture. Accurate information about the molecular weight, discrimination between isoforms and possible capture of co-precipitating proteins, represent some of the most attractive information that could be obtained by using this technology.

MCP-4/CCL13 is a chemokine whose concentration in the blood is in the range of 200–300 pg/ml [3,7]. This protein is generally easily detected and quantified by ELISA but not by Western Blot analysis [11]. Matrix assisted laser desorption mass spectrometry (MALDI) analysis of purified recombinant human MCP-4/CCL13 expressed in insect revealed a predominant peak with a mass of about 8575 m/z, which starts with residue 24at QPDAL. Two minor peaks at about 8760 and 9314 m/z, starting at residue 22 and 17 respectively were also detected. The alignment of MCP-4/CCL13 sequence with those of well characterized members of the CC-chemokine family strongly suggested that the recombinant form of m/z 8575, synthesized by *Drosophila *cells, is the correct mature protein [11,12]. Alternative splicing has not been found [13]. To date, human MCP-4/CCL13 has not yet been purified from serum or other human fluid/tissue and no evidence of naturally occurring "isoforms" have been reported. By using SELDI immunoaffinity capture chip technology we were able to identify two MCP-4/CCL13 isoforms and evaluate their molecular weight in a complex protein mixture such as serum. The molecular weights of the isoforms we detected in the serum do not correspond to those previously described for the recombinant protein. We detected a major peak at m/z of 8945 instead of 8575, and a minor peak of a m/z of 9113 instead of 8760. The apparent molecular weight of the major serum component is consistent with a peptide starting at residue 20 and extending to residue 98 of the amino acid sequence deduced from cDNA. Thus it would appear possible that the signal peptide is 19 amino acids and not the 16 previously postulated from the recombinant forms (UniProtKB/SWISS-Pro-entry-Q996126) [15]. Three possible phosphorylation sites (S 44, S 50, T 55) are found in the MCP-4/CCL13 sequence when analyzed by NetPhos 2.0 Server (EXPASY database ), these are the only post translational modifications potentially occurring given the MCP-4/CCL13 amino acid sequence. It is possible that some of the differences between the isoforms we detected and those observed in the recombinant protein are the result of phosphorylation. We cannot exclude post-translational modification of the serum MCP-4/CCL13 nor speculate on the identity of our 9113 M/Z component. Our assessment of MCP-4/CCL13 isoforms by SELDI immunocapture was purely qualitative (differential quantification between pre and post treatment samples was not consistent). We postulated that in our experimental condition, saturation of antibody crosslinked to the MCP-4/CCL13 protein on the SELDI chip was the limiting factor as previously described by Hess et al. [10]. In conclusion, this short report establishes for the first time the occurrence of two different isoforms of human MCP-4/CCL13 in the peripheral blood. Furthermore, it demonstrates the versatility of SELDI in rapidly detecting MCP-4/CCL13 isoforms.
